# Gut‐on‐a‐chip platforms: Bridging in vitro and in vivo models for advanced gastrointestinal research

**DOI:** 10.14814/phy2.70356

**Published:** 2025-05-05

**Authors:** Awurama Ofori‐Kwafo, Indira Sigdel, Earshed Al Mamun, Jasenka Zubcevic, Yuan Tang

**Affiliations:** ^1^ Department of Bioengineering, College of Engineering University of Toledo Toledo Ohio USA; ^2^ University of South Florida Center for Microbiome Research Microbiomes Institute Tampa Florida USA; ^3^ Department of Neurosurgery and Brain Repair University of South Florida Morsani College of Medicine Tampa Florida USA

**Keywords:** gastrointestinal tract, gut‐brain axis, gut‐on‐a‐chip, intestinal models, microbiome

## Abstract

The gastrointestinal (GI) tract plays a critical role in nutrient absorption, immune responses, and overall health. Traditional models such as two‐dimensional cell cultures have provided valuable insights but fail to replicate the dynamic and complex microenvironment of the human gut. Gut‐on‐a‐chip platforms, which incorporate cells located in the gut into microfluidic devices that simulate peristaltic motion and fluid flow, represent a significant advancement in modeling GI physiology and diseases. This review discusses the evolution of gut‐on‐a‐chip technology, from simple cellular mono‐cultures models to more sophisticated systems incorporating bi‐cultures and tri‐cultures that enable studies of drug metabolism, disease modeling, and gut–microbiome interactions. Although challenges remain, including maintaining long‐term cell viability and replicating immune responses, these platforms hold great potential for advancing personalized medicine and improving drug discovery efforts targeting gastrointestinal disorders.

## 1 INTRODUCTION

### 1.1 Structure and function of the gastrointestinal tract

The gastrointestinal (GI) tract, or the digestive system, includes a series of organs beginning with the mouth and extending through the esophagus to the stomach and small and large intestine. Its primary role is in food digestion, absorption of nutrients, and expelling waste (FitzPatrick & Keshav, 2020; McQuilken, 2021; Sauer & Merchant, 2018). However, the GI tract has other important roles, including its involvement in the hosts' immunity as well as the etiopathology of several disorders within and beyond the GI tract (Kong et al., 2018; Morgan et al., 2012; Moser et al., 2018; Nagatake et al., 2019; Senda et al., 2019).

**FIGURE 1:**
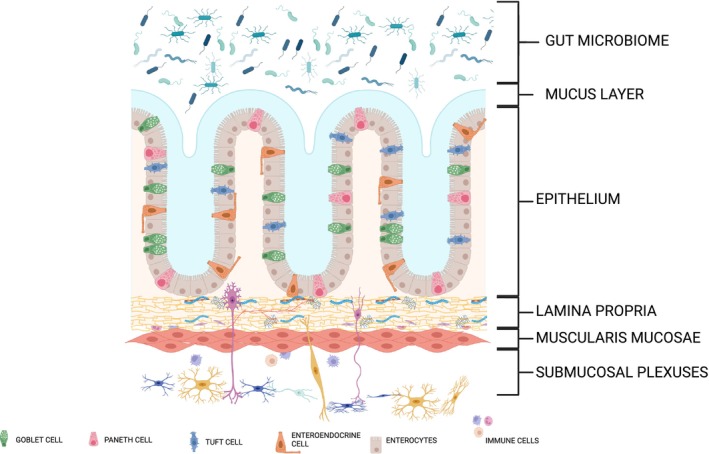
Structural organization of the gastrointestinal layers and their functional components. The simplified schematic of a cross section of the gastrointestinal tract illustrates the intestinal structural organization including the gut microbiome at the surface to the submucosal plexus. The mucus layer acts as a barrier against pathogens, with the outer layer housing gut microbes. The epithelium consists of several specialized cells, including goblet cells (mucus‐secreting), Paneth cells (antimicrobial), tuft cells (chemosensory), enteroendocrine cells (hormone‐secreting), and enterocytes (nutrient absorption). Beneath this, the lamina propria contains connective tissue, blood vessels, and immune cells, supporting both structure and immune function. The muscularis mucosae facilitates movement of the mucosa, while the submucosal plexuses regulate blood flow, secretions, and gut motility. Created in BioRender. Tang, Y. (2025) BioRender.com/e36e690.

The gut comprises several layers. The mucosal layer consists of three main sub‐layers, including the epithelium, the lamina propria, and the muscularis mucosae (Hall, 2013) (Figure 1). The epithelium, the innermost layer of the mucosa, consists of various cell types (Figure 1), including enterocytes for nutrient absorption, goblet cells for mucus secretion, several types of enteroendocrine cells involved in the secretion of peptides and hormones, tuft cells involved in chemo sensing and immune signaling, and Paneth cells, in part, involved in antimicrobial peptide secretion (Kurashima & Kiyono, 2017). It also acts as a physical barrier to prevent the entry of harmful pathogens and allows the passage of ions and water, short‐chain fatty acids (SCFAs) and other bacterial metabolites (Odenwald & Turner, 2017). The link between GI disorders and the disruption of the intestinal barrier underscores the importance of acquiring a thorough comprehension of its function (Di Tommaso et al., 2021; Salim & Söderholm, 2010).

The mucus layer serves as a protective barrier over the epithelium, guarding against pathogens. The inner layer, which is epithelium‐bound and defends against microbial invasion, and the outer layer, the less dense unattached layer that provides a habitat for the gut microbiome, facilitates gut homeostasis (Fleming et al., 2020; Spencer & Hu, 2020). The microbiome is a uniquely diverse community of microorganisms in the GI tract that has emerged as a key player in host physiology, including but not limited to digestion, metabolism, blood pressure regulation, immune, and neural function (Gibbons, 2019; Loh et al., 2024; Ullah et al., 2023; Wehrwein et al., 2016; Yang et al., 2015). Examining the complexity of the gut microbiome in a regulated, dynamic microenvironment will aid in the identification of new mechanisms that contribute to the disruption of the microbiome environment and facilitate the creation of new treatments for gut microbiome‐related ailments (Durack & Lynch, 2019). Supporting the GI epithelium is the lamina propria, composed of loose connective tissue, blood vessels, and immune cells (Figure 1) (Targan, 1992). The outermost layer, the muscularis mucosae, comprises a thin layer of smooth muscle fibers that facilitate the movement and folding of the mucosal layer (Hall, 2013).

### 1.2 The enteric nervous system and the gut‐brain axis

Beneath the mucosa is the submucosa, consisting of dense connective tissue, blood vessels, and the submucosal plexus, a network of autonomic nerves regulating local blood flow, secretions, and absorption in the mucosa (Figure 1) (Van Eyken et al., 2014). The submucosal and myenteric plexus constitute the enteric nervous system, a complex network of 200–600 million neurons situated in the GI tract (Furness et al., 2014; Goldstein et al., 2013). The enteric nervous system regulates GI motility, secretion, blood flow, and immunity. It operates autonomously while also working in conjunction with the central nervous system to ensure the efficient functioning of the digestive system and the maintenance of homeostasis in the GI tract (Browning & Travagli, 2014).

This connection is commonly termed the gut‐brain axis (GBA) and encompasses not just anatomical neural connections, but also includes endocrine, humoral, metabolic, and immunological communication pathways (Appleton, 2018; Kuwahara et al., 2020). The vagus nerve serves as the primary neuronal pathway that facilitates communication between the gut and the brain. This intricate communication system between the gut and the brain is crucial for maintaining homeostasis and regulating various physiological processes in the human body (Cryan et al., 2019; de Araujo et al., 2024; Gheorghe et al., 2019; Margolis et al., 2021). The study of this bidirectional communication is critical to comprehending various disease mechanisms and has been at the forefront of recent research developments (Bonaz et al., 2017; Cryan et al., 2019; de Araujo et al., 2024; Liu et al., 2015).

### 1.3 The role of in vivo and in vitro models in GI research

Understanding the gut–microbiota interactions and brain–body connections can lead to breakthroughs in treatments and preventive measures for numerous health conditions, thus improving quality of life and longevity, but significant gaps remain in our understanding of the mechanisms behind its influence. These gaps arise from inadequate study methods and the limited availability of effective models outside in vivo. In vivo models offer an advantage for multisystem evaluation of the role of the GI in physiological homeostasis, making them crucial for drug development and testing therapeutic strategies. The role of microbiota in intestinal vagal signaling has been investigated in hypertension in vivo using calcium imaging in vagal afferent neurons projecting to the gut (de Araujo et al., 2024) and numerous studies have used this approach to investigate the role of the vagus in physiologic homeostasis (Baumer‐Harrison et al., 2024; Kim et al., 2020). Others have used in vivo genetic manipulation of vagal activity or nerve dissection to investigate the role of the gut in behavior, metabolism, neurologic conditions, and learning and memory (Bravo et al., 2011; Brierley et al., 2021; Davis et al., 2020; McDougle et al., 2024). However, results from animal models may not fully translate to humans due to differences in physiology and microbiome composition, necessitating further research to validate the findings in human systems. The challenges, particularly the complexity of interpreting results, underscore the limitations of in vivo models and highlight the growing need for in vitro and ex vivo alternatives.

Beyond animal models, in vitro models have been used to investigate biological processes in a highly controlled setting and are pivotal in understanding fundamental mechanisms (Mainville et al., 2005; McCright et al., 2022). Ideally, in vitro systems should closely mimic in vivo conditions and replicate complex cellular interactions and physiological environments, thereby enhancing their relevance and accuracy in predicting biological responses and enabling the translational potential of findings from animals to humans (Herrmann et al., 2014; Ramadan & Gijs, 2015). These systems reduce complexity and minimize animal usage by offering a cost‐effective alternative to traditional in vivo studies. They enable high‐throughput screening of drug candidates, toxicological assessments, and detailed mechanistic studies (Astashkina et al., 2012). Additionally, they act as a critical translational bridge, bridging the gap between animal research and human investigation, thus reducing reliance on animal models while providing more human‐relevant data (Carvalho et al., 2023; Konishi et al., 2015; Wu et al., 2023; Xiang et al., 2020).

### 1.4 Organ‐on‐a‐chip technology

In recent years, organ‐on‐a‐chip technology has emerged as an essential tool to bridge the gap between in vivo and in vitro models (Ramadan & Zourob, 2020). By mimicking the multicellular architectures, cell to cell interfaces, physicochemical microenvironments, and vascular perfusion of the body, organ‐on‐a‐chip devices provide an advanced level of in vitro simulations (Bhatia & Ingber, 2014). These devices enable high‐resolution, real‐time imaging and in vitro analysis of biochemical, genetic, and metabolic activities of living cells. The gut on a chip, which is the term used to refer to replicating the gut microenvironment in an organ‐on‐a‐chip system, mimics the structure and function of the mammalian intestine for the study of the complex cell to cell interactions and precise manipulation of conditions and variables that can inform in vivo and clinical studies (Marrero et al., 2021; Xiang et al., 2020). They offer a unique opportunity to explore the nuanced relationships in the GI environment (Ashammakhi et al., 2020; Garcia‐Gutierrez & Cotter, 2022; Valiei et al., 2023). Significant advancements have been made in gut‐on‐a‐chip models recently, evolving from simple cellular mono‐cultures systems to more complex bi‐culture and tri‐culture setups. As these models have developed, additional functionalities including various mechanical simulations have been introduced to increase their physiological relevance, allowing them to more accurately reflect in vivo conditions. These improvements have enabled more precise modeling of physiological processes and disease states, thereby bringing gut‐on‐a‐chip systems closer to replicating the complexities of the mammalian GI environment (Jing et al., 2022; Kim et al., 2012; Pöschl et al., 2023).

### 1.5 Advancements and challenges in gut‐on‐a‐chip models

Current gut‐on‐a‐chip models have successfully incorporated key physiological attributes such as diverse epithelial cell populations, functional mucus layers, and mechanical cues like peristalsis‐mimicking to simulate intestinal motility (Baldwin et al., 2023; Villenave et al., 2017). These systems have also integrated microbial communities to study host–microbe interactions, coculture endothelial or immune cells for basic immune‐epithelial crosstalk, and incorporated emerging enteric nervous system components for gut‐brain axis exploration, alongside real‐time monitoring of barrier integrity and drug metabolism (Beaurivage et al., 2020; de Hoyos‐Vega et al., 2023; Jalili‐Firoozinezhad et al., 2018; Kim et al., 2012). However, critical gaps remain that hinder their full potential in drug discovery. These include the lack of comprehensive immune system features, incomplete microbiome diversity and spatial heterogeneity, and limited systemic multi‐organ interactions. Addressing these challenges through advanced immune modeling and multi‐organ integration will improve their ability to predict drug efficacy, toxicity, and microbiome‐mediated responses, ultimately bridging the gap between preclinical models and human trials.

This review examines the evolution of gut‐on‐a‐chip models, tracing their progression from cellular mono‐culture systems to advanced bi‐ and tri‐culture designs that incorporate epithelial, microbial, immune, and neural components. We then explore their applications in drug discovery, including oral drug absorption studies, microbiome‐targeted therapeutic screening, and gut‐brain axis research, highlighting how these models address gaps in traditional in vitro and in vivo approaches.

We also analyze the physiological relevance of gut‐on‐a‐chip models in replicating host–microbiota interactions, mechanical cues, and disease microenvironments, while addressing limitations such as incomplete immune system modeling and scalability challenges. Finally, we discuss future directions, including integration with multi‐organ systems, patient‐specific customization, and advancements in chronic disease modeling. By contextualizing these developments within the broader landscape of biomedical research, this review aims to guide researchers in leveraging gut‐on‐a‐chip platforms to overcome translational barriers and accelerate therapeutic innovation.

## 2 CELLULAR MONO‐CULTURES

**FIGURE 2:**
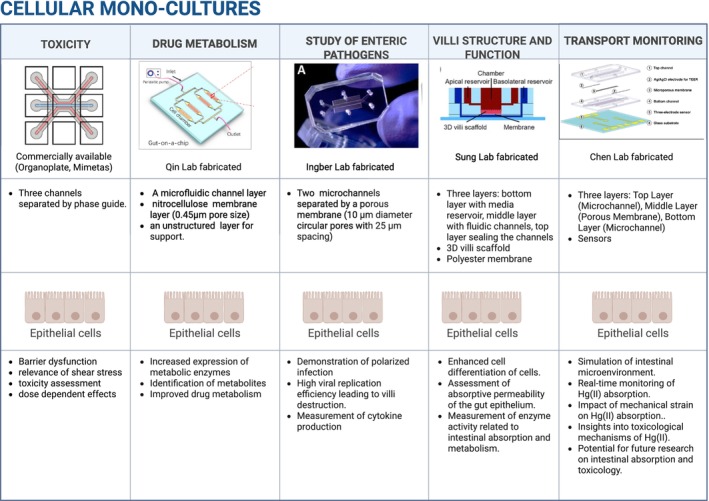
Cellular mono‐cultures culture platforms using epithelial cells replicate in vivo conditions for specialized research applications. The figure summarizes the applications of cellular mono‐cultures culture systems in five key research areas: Toxicity, drug metabolism, the study of enteric pathogens, villi structure and function, and transport monitoring. Each column includes a schematic representation of the system setup, details of its structure, and the functional outcomes. Across all applications, epithelial cells are the primary cell type used. The systems vary in design, including multi‐layer setups with porous membranes, microchannels, and 3D villi scaffolds, enabling a range of studies such as toxin response, drug metabolism, pathogen infection, nutrient absorption, and transport processes (Guo et al., 2018; Pöschl et al., 2023; Shim et al., 2017; Villenave et al., 2017; Wang et al., 2023). Created in BioRender. Tang, Y. (2025) https://BioRender.com/y53m745. Figure of devices reproduced from Pöschl et al. (2023); Guo et al. (2018); Villenave et al. (2017); Shim et al. (2017); Wang et al. (2023).

Cellular mono‐cultures in microfluidic devices represent an early stage in the development of gut‐on‐a‐chip models and have provided valuable insights into cellular function in controlled environments by making this the first step in recreating precisely controlled relevant physiological microenvironments (Figure 2) (Chao & Ros, 2008; Guo et al., 2018; Pöschl et al., 2023; Villenave et al., 2017; Wang et al., 2023).

Compared to organoids, microfluidic devices offer a more structured and controllable environment, allowing for the regulation of mechanical forces like stress and peristalsis‐like motion and precise nutrient flow (Kim et al., 2012). This design facilitates improved access to both apical and basolateral compartments and enables real‐time monitoring of biological responses, features that are challenging to achieve in traditional organoid cultures (Liu et al., 2024; Sidar et al., 2019; Valiei et al., 2023).

The cells widely used for organ‐on‐a‐chip studies of the GI tract are the human colon adenocarcinoma cells, (Caco‐2 cells) due to their ability to mimic the human intestinal conditions (Sambuy et al., 2005). Caco‐2 cells possess both morphological and functional characteristics of enterocytes, making them a valuable model for studying the intestinal microenvironment (Sambuy et al., 2005). In addition, they exhibit brush border microvilli, a characteristic in the intestinal layer that enhances surface absorption (Sambuy et al., 2005). Tight junctions are formed in the Caco‐2 cells, a key indication of intestinal barrier integrity, and can also control the movement of substances between cells in the gut (Iftikhar et al., 2020; Sambuy et al., 2005). Additionally, they have been utilized as predictive models to examine oral drug absorption via transport mechanisms in the human intestinal mucosa and have served as a robust model for studying intestinal barrier integrity and permeability (Artursson et al., 2012; Hilgers et al., 1990; Iftikhar et al., 2020). Caco‐2 cells can be cultured under numerous conditions and can produce consistent results, making them an invaluable tool in modeling the human intestine on a chip (Sambuy et al., 2005).

However, while Caco‐2 cells possess select properties of intestinal epithelial cells, they are derived from colon cancer cells and may not fully replicate the complex functions of the small intestine (van Breemen & Li, 2005). Unlike the in vivo environment, they lack a functional mucus layer unless cocultured with mucus‐secreting cell lines (Hu et al., 2022). Additionally, they exhibit low levels of metabolic enzymes such as CYP3A4 and overexpress transporters like P‐glycoprotein, which can lead to inaccurate predictions of drug metabolism and absorption (Wilson et al., 1990). These limitations hinder their ability to fully replicate the complex functions of the intestinal epithelium, particularly in studies related to drug permeability, metabolism, and host–microbiota interactions.

To overcome these limitations, primary intestinal epithelial cells present a more physiologically relevant alternative for gut‐on‐a‐chip models. These cells retain the diverse functional properties of the human gut, including mucus secretion, active metabolic enzyme expression, and a more representative transporter profile, making them better suited for studying drug absorption, host–microbe interactions, and disease modeling in a biologically relevant manner (Li et al., 2022; Pimenta et al., 2022; Verhulsel et al., 2020).

### 2.1 Toxicity

Utilizing a cellular mono‐cultures culture setup, one study sought to examine the impact of deoxynivalenol, a common mycotoxin found in human food, on intestinal barrier function using a novel three‐dimensional gut‐on‐a‐chip model (Pöschl et al., 2023). This advanced model incorporated physiologically relevant conditions including intestinal flow, thereby providing a more accurate representation of the in vivo environment. Previous research on deoxynivalenol has primarily relied on animal models and conventional two‐dimensional cell cultures, which lack complexity and relevance to human physiology, often leading to potential inaccuracies (Diesing et al., 2011; Kasuga et al., 1998; Pestka et al., 2008).

The Caco‐2 cells were cultured in a commercially available microfluidic platform called the Organoplate, which consists of 40 microfluidic chips arranged in a 384‐well plate configuration, each with three channels and an observation window for real‐time monitoring of the three‐dimensional cultures (Figure 2). By placing the Organoplate on a rocker that tilted at a 7° angle every 8 min, they simulated shear stress to mimic physiological conditions, thus incorporating flow to better understand the complex interactions between mycotoxins and the intestinal barrier and providing a more accurate representation of the human intestinal barrier compared to traditional static cell cultures. Assays such as transepithelial electrical resistance and barrier integrity assessments were used to evaluate the effects of the toxin. This highlighted the potential of microfluidic models in toxicological research, paving the way for future studies on antitoxic compounds (Pöschl et al., 2023).

The three‐dimensional microfluidic system outperformed conventional two‐dimensional static cultures by incorporating fluid flow and shear stress, which enhanced intestinal barrier function, as evidenced by higher transepithelial electrical resistance values and improved tight junction integrity. Additionally, its real‐time monitoring capability enabled continuous assessment of barrier integrity and toxin effects, providing more accurate and dynamic insights (Pöschl et al., 2023). These findings highlight the need for further integration of biomechanical forces and continuous monitoring in gut‐on‐a‐chip models to improve their predictive power for drug testing and disease modeling.

#### 2.1.1 The potential and limitations of cellular mono‐cultures models

Cellular mono‐cultures models have played a vital role in foundational research, especially in isolated studies of cellular functions, drug absorption, and epithelial barrier properties (Guo et al., 2018; Pöschl et al., 2023; Shim et al., 2017). These models provide a simplified and controlled environment that is particularly valuable for early‐stage research, where isolating the effects of a cellular mono‐cultures type is necessary (Chen et al., 2012). However, the limitations of cellular mono‐cultures models stem from their inherent oversimplification. They cannot replicate the complex interactions and structures in the GI environment (Duell et al., 2011). The lack of cellular diversity restricts their ability to accurately represent in vivo physiological processes, such as immune responses, cell–cell communication, and host–microbiota interactions, critical for understanding numerous diseases and physiological conditions (Astashkina et al., 2012; Choi et al., 2014; de Araujo et al., 2024; Yang et al., 2015). The need for more advanced and complex models and the addition of microbiome is essential for improving the accuracy of scientific findings.

Replicating in vivo physiological processes, such as immune responses, cell–cell communication, and host–microbiota interactions, is crucial for studying several diseases and physiological conditions. The interaction between gut epithelial cells and immune cells is critical in gastrointestinal disorders, and utilizing more advanced bi‐ and tri‐ culture models incorporating immune components helps assess inflammation and therapeutic responses more accurately (Jing et al., 2022).

### 2.2 Drug metabolism

Intestinal drug metabolism can significantly reduce bioavailability, accounting for more than 20% of overall first‐pass metabolism (George, 1981; Kato et al., 2003; Varma, 2010) and gut‐on‐a‐chip models can be used to gain a more comprehensive understanding of these processes (Guo et al., 2018). One such model (Figure 2) incorporated a porous nitrocellulose membrane and collagen I (Guo et al., 2018) thereby establishing a three‐dimensional microenvironment for Caco‐2 epithelial cells and allowing for continuous media perfusion to ensure efficient nutrient delivery, oxygenation, and waste removal. By simulating the dynamic conditions of the intestinal environment, this configuration offered a more accurate representation of physiological processes. The functionality of Caco‐2 cells in this model was evaluated by assessing mucus secretion by alcian blue staining, while gene expression analysis measured differentiation‐related changes in intestinal gene expression. Cells were treated with ifosfamide and verapamil, and the supernatant was analyzed to assess their metabolism. The biomimetic intestinal microenvironment, with its constant flow and three‐dimensional porous membrane, facilitated the formation of a compact epithelial layer and promoted increased expression of cytochrome P450 enzymes, which play a critical role in drug metabolism (Guo et al., 2018).

However, beyond CYP450 enzymes, the gut is also involved in phase II metabolism and microbial metabolism, influencing drug efficacy, toxicity, and individual responses. Understanding these processes is critical for drug development, personalized medicine, and gut health research (Basit et al., 2020; Ervin et al., 2020; Swanson, 2015). The human gut contains unique enzymes, including microbial enzymes, which impact how drugs and other compounds are processed, making it essential to develop models that accurately mimic human gut metabolism (Javdan et al., 2020; Sharma et al., 2017). One major limitation of current models is species differences in metabolism. Human‐specific enzymes are poorly replicated in animals, leading to inaccurate drug metabolism predictions (Basit et al., 2022).

A gut‐on‐a‐chip model incorporating human‐derived cells and enzymes can help address these challenges by providing a physiologically relevant platform to study host‐microbe interactions, phase II metabolism, and interindividual variability, improving drug development and precision medicine.

### 2.3 Study of enteric pathogens

Villenave, Wales et al. investigated the polarized infection of Coxsackie B1 virus using a gut‐on‐a‐chip model (Villenave et al., 2017). Coxsackie B is a known GI pathogen that can lead to myocarditis and viral meningitis (Pasch & Frey, 2006). Human Caco‐2 cells were cultured in the device, forming three‐dimensional villi‐like structures characteristic of the small intestine (Figure 2). The virus was introduced onto either the apical or basolateral surfaces of the polarized intestinal cells, allowing the study of polarized infection and its effects on the epithelium (Villenave et al., 2017).

Initially, coxsackievirus B1 (CVB1) was introduced into the gut chip by adding a specific volume of stock virus to either the apical or basal channels of the device. After allowing the virus to adsorb for 2 h under static conditions, the unbound virus was washed away, and the chips were put back under flow conditions to simulate physiological environments. To monitor the infection over time, apical and basal effluents were collected at various time points (6, 24, and 48 h post‐infection) (Villenave et al., 2017).

The platform provided continuous fluid flow, replicating the dynamic environment of the intestine and facilitating the spread of newly formed virions and secondary infections across the epithelium. The infection process was observed in real time, allowing researchers to monitor cytopathic effects and villus destruction. The model enabled the study of viruses that are difficult to culture using traditional methods, offering insights into viral replication, release, and infection mechanisms within the human intestinal epithelium. The microfluidic design of the gut‐on‐a‐chip allowed for the continuous collection of effluents and real‐time monitoring of viral replication, cytokine production, and cellular responses. This capability allowed temporal tracking of infection dynamics and host responses. The findings contributed to a deeper understanding of enteric virus biology, host‐pathogen interactions, and the immune response in the gut. Future mechanistic studies and antiviral strategies using this model may provide a useful tool for researchers in virology and gastroenterology (Villenave et al., 2017).

### 2.4 Villi structure and function

Accurately representing gut structure is vital for advancing and assessing new medications aimed at treating GI disorders in a biologically relevant setting for the development of efficient and targeted therapies.

Many new medications rely on physiologically relevant villi structures for accurate testing and development, including oral biologics, which require villi models to assess absorption and stability (Taebnia et al., 2021). Additionally, targeted drug delivery systems for inflammatory bowel diseases also benefit from such models to optimize absorption and minimize side effects (Yi et al., 2017).

One study was motivated by the need for improved experimental models that better replicate the complexities of the human gut, with the goal of enhancing our understanding of intestinal villi, which have important implications for drug development and disease research (Ensari & Marsh, 2018; Shim et al., 2017). Caco‐2 cells were cultured either as a two‐dimensional monolayer on a porous membrane or in a three‐dimensional form on a collagen villi scaffold within a microfluidic device to replicate the intestinal villi structures (Figure 2) (Shim et al., 2017). The villi scaffold provided a larger surface area for cell attachment and growth, enhancing the model's physiological relevance. The study combined fluidic shear stress with a three‐dimensional culture environment, an approach that reflects the dynamic conditions of the gut, where cells experience both mechanical forces from fluid flow and structural complexity. A variety of techniques were used to assess multiple aspects of gut cell physiology, including cell morphology, cytochrome P450 3A4 activity, and permeability assays. This comprehensive approach provided a holistic view of how the three‐dimensional cultures and fluidic environment influence gut cell function, a perspective often lacking in cellular mono‐cultures layer models, as the microenvironment significantly influenced the morphology, enzyme activity, and permeability characteristics of the gut epithelium (Shim et al., 2017).

### 2.5 Real‐time monitoring of transport and absorption

Earlier studies on gut‐on‐a‐chip models primarily focused on replicating the intricate structural features of the human intestine, including the three‐dimensional villi morphology and brush border microvilli with tight junctions (Kim & Ingber, 2013; Yu et al., 2012). However, more recent research has shifted towards investigating functional phenomena such as transport processes. One such cellular mono‐cultures study used a gut‐on‐a‐chip model to address significant gaps in understanding of intestinal physiology, particularly in relation to the transport mechanisms of toxic substances such as mercury (Hg(II)). In this study, Caco‐2 cells were used to recreate the intestinal environment within the chip, which was equipped with embedded sensors to monitor the transport of Hg(II) in real time (Figure 2) (Wang et al., 2023). The study integrated advanced microfluidic technology and label‐free sensors to enable real‐time monitoring of cellular responses and transport processes. As in most gut‐on‐a‐chip models, the device consisted of top and bottom microchannels separated by a porous membrane. Transepithelial electrical resistance measurements were performed using integrated Ag/AgCl electrodes to monitor the integrity of the cell monolayer. Electrochemical impedance spectroscopy recorded changes in resistance, and apparent permeability coefficients were calculated to evaluate Hg(II) transport across the cell layer. The incorporation of sensors and electrochemical detection enabled real‐time, noninvasive monitoring of intestinal barrier integrity and Hg(II) absorption. This dynamic observation provided a significant advantage over traditional static culture models and animal studies, allowing for more precise investigation of transport mechanisms and toxicological effects on intestinal physiology. By simulating the intestinal barrier and monitoring the transport of Hg(II), the model serves as a valuable tool for studying the functional aspects of the intestine in vitro (Wang et al., 2023).

## 3 BI‐CULTURE

The oversimplified nature of cellular mono‐cultures limits their ability to replicate key physiological processes, such as communication between multiple cell types, immune responses, and overall tissue function (Dura et al., 2016; Hong et al., 2012).

This limitation is particularly significant in studying physiological and pathological conditions where complex interactions drive disease progression. For example, inflammatory bowel diseases such as Crohn's disease and ulcerative colitis rely on immune–epithelial interactions that regulate inflammation and barrier function (Campbell & Colgan, 2017). Disruption of epithelial integrity in these diseases leads to increased permeability, immune activation, and chronic inflammation (Bradford et al., 2012). Additionally, epithelial‐microbe interactions play a crucial role, as the gut microbiota influences immune responses, inflammation, and tissue homeostasis (Jergens et al., 2021). These complexities underscore the need for advanced gut‐on‐a‐chip models that integrate immune components and microbiota to better replicate the in vivo disease environment and improve drug testing outcomes.

**FIGURE 3:**
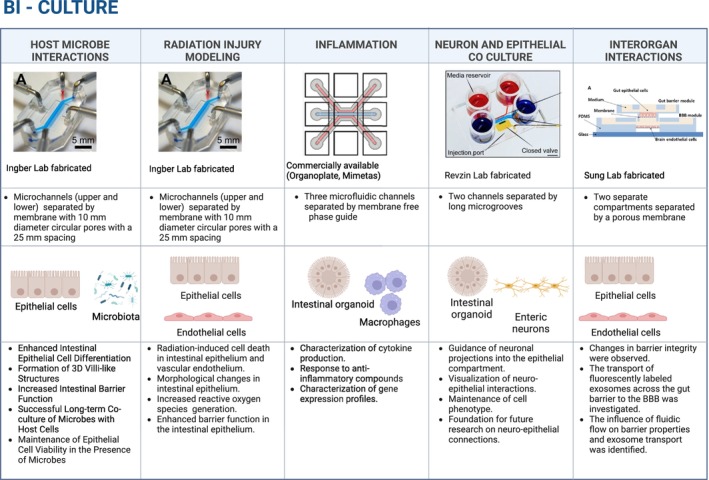
Bi‐culture gut‐on‐a‐chip platforms replicate in vivo conditions by incorporating advanced device setups. The figure summarizes the applications of bi‐culture systems in five research areas: Host–microbe interactions, radiation injury modeling, inflammation, neuron and epithelial coculture, and interorgan interactions. Each system employs distinct setups, including microchannels, porous membranes, phase guides, or compartmentalized chambers, with epithelial cells, intestinal organoids, and enteric neurons as the primary cell types. Key applications include studying epithelial differentiation, intestinal barrier function, and coculture with microbes; analyzing radiation‐induced damage and oxidative stress; characterizing cytokine production and gene expression; exploring neuroepithelial interactions and neuronal guidance; and investigating barrier integrity, exosome transport, and interorgan communication( Kim et al. (2012); Jalili‐Firoozinezhad et al. (2018); Beaurivage et al. ( 2020); de Hoyos‐Vega et al. (2023); Kim et al.(2021). Created in BioRender. Tang, Y. (2025) https://BioRender.com/y53m745. Figure of devices reproduced from Kim et al. (2012); Jalili‐Firoozinezhad et al. (2018); Beaurivage et al. (2020); de Hoyos‐Vega et al. (2023); Kim et al. (2021).

To address these limitations, researchers have developed more advanced bi‐culture models that incorporate interactions between two cell types (Figure 3) (Duell et al., 2011; de Haan et al., 2021; Kim et al., 2012; Rogers et al., 2021). This enhanced complexity allows for a more accurate examination of key physiological processes that are not fully captured by cellular mono‐cultures (Van den Abbeele et al., 2010; Rogers et al., 2021).

A significant step forward in gut‐on‐a‐chip studies is the utilization of primary epithelial cells, which are commonly cocultured in advanced models. Derived directly from tissue samples, these cells preserve the functional characteristics of their tissue of origin, exhibiting characteristics such as barrier function, polarity, and interactions with the microbiome, essential for understanding intestinal health and disease and making them ideal for studying cellular and molecular mechanisms in the GI tract (Kaeffer, 2002; Zhou et al., 2022). These cells also hold promise for personalized medicine, as they can be derived from specific patients to investigate disease mechanisms and test therapeutic approaches (Maddipatla et al., 2022; Shilova et al., 2022). Despite their utility, primary epithelial cell cultures also come with certain challenges. Isolating and maintaining the cells require specialized expertise, while donor variability can lead to inconsistencies in experimental outcomes, thus potentially complicating reproducibility and hindering the generalization of findings (Kaeffer, 2002). In the following sections, we review the advantages and limitations of these models in greater detail.

### 3.1 Host–microbe interactions

One study marks a significant advancement in integrating microbiota into gut‐on‐a‐chip models. The gut microbiota plays a crucial role in maintaining homeostasis through the breakdown of complex carbohydrates, fibers, and other nutrients indigestible by human enzymes (Hansen & Sams, 2018) Host–microbiome interactions play a crucial role in enhancing gut absorption by facilitating the breakdown of complex carbohydrates and fibers that human enzymes cannot fully digest, thus producing short‐chain fatty acids that the host can utilize for energy (Krajmalnik‐Brown et al., 2012). It contributes to the development and maturation of immune cells, maintains immune tolerance to harmless antigens, and protects against pathogens by competing for resources and producing antimicrobial compounds (Shi et al., 2017; Wan et al., 2019; Wu & Wu, 2012). The microbiome in the gut aids in nutrient absorption and vitamin synthesis and produces short‐chain fatty acids critical for colonic health (Pushpanathan et al., 2019; Silva et al., 2020). Its influence extends beyond the gut, affecting cardiovascular, metabolic, immune, neural, and behavioral outcomes through the production of bioactive molecules, neurotransmitters, and metabolites (Bu & Wang, 2018; Manco et al., 2010; Strandwitz, 2018; Tang et al., 2017). A balanced microbiome is vital for overall homeostasis, and an imbalance known as dysbiosis has been linked to various diseases and conditions, including but not limited to diabetes, metabolic syndrome, and cardiovascular diseases (Agus et al., 2021; Cuevas‐Sierra et al., 2019; Dieterich et al., 2018; Festi et al., 2014; Guinane & Cotter, 2013; Hou et al., 2022; Meijnikman et al., 2018; Muñoz‐Garach et al., 2016; Tang et al., 2017).

One study successfully demonstrated the coculture of *Lactobacillus rhamnosus* GG with Caco‐2 cells for over 14 days, preserving cell viability by recreating peristaltic motions and oxygen gradients (Kim et al., 2012). *Lactobacillus rhamnosus* GG was used in the gut‐on‐a‐chip model for its intestinal barrier enhancing function and to modulate the immune responses (Kim et al., 2012). The model enabled the culture of human intestinal epithelial cells with microbial flora in the presence of physiologically relevant luminal flow and peristalsis‐like mechanical strain (Kim et al., 2012). The microdevice used in this study consisted of two channels separated by a polydimethylsiloxane membrane with 10 μm circular pores (Figure 3). The Caco‐2 cell line simulated the intestinal layer, and a computer‐controlled system applied repetitive suction to mimic peristaltic movements. This coculture system promoted the differentiation of intestinal epithelial cells, the formation of three‐dimensional villi‐like structures, and improved intestinal barrier function under simulated gut conditions. The application of cyclic mechanical strain, mimicking peristalsis, underscored the importance of mechanical forces in regulating intestinal cell activity. This model allowed for the mechano‐regulation of intestinal functions to be studied, providing insight into how physical forces impact gut health and disease. Incorporating *Lactobacillus rhamnosus* GG into the model allowed for a more realistic simulation of natural interactions between probiotics and host cells within the gut environment, enhancing its physiological relevance. Additionally, the incorporation of peristaltic motions and fluid flow further improved the model's representation of intestinal functions, offering a more accurate alternative to static in vitro models. Together, these features contribute to a model with heightened physiological accuracy (Kim et al., 2012).

#### 3.1.1 The potential and limitations of bi‐culture models

Bi‐culture models, incorporating two distinct cell types, advance research by enabling more complex physiologically relevant interactions. They aid in studying nutrient absorption, barrier function, immune responses, and other functions in health and GI conditions that rely on cellular interactions. They have been particularly useful in exploring epithelial–immune interactions, epithelial‐endothelial crosstalk, and microbiome–host interactions in controlled settings.

While bi‐culture models offer valuable insights and a more physiological approach to cellular mono‐culture systems, they fall short in replicating the full complexity of the gut microenvironment. For example, in inflammatory bowel disease research, the absence of a fully integrated immune component including macrophages, dendritic cells, and T cells restricts the model's ability to capture complex immune–epithelial interactions that drive disease progression (Beaurivage et al., 2020). Additionally, microbiome–host interaction studies are constrained by the lack of a fully functional mucus layer and diverse microbial populations, which are critical for studying bacterial colonization, metabolic interactions, and dysbiosis‐related diseases (Kim et al., 2012).

These limitations highlight the need for more advanced multicellular models that integrate epithelial, immune, neural, and microbial components to better mimic in vivo gut complexity. By incorporating these elements, future gut‐on‐a‐chip models will enhance their physiological relevance for applications in drug development, disease modeling, and personalized medicine.

### 3.2 Modeling radiation injury‐induced cell death

In another bi‐culture advancement, one study investigated the effects of radiation exposure on the GI. This study investigated the effects of radiation exposure on the GI structure and function, particularly focusing on the intestinal epithelium and vascular endothelium, including aspects such as villus morphology, barrier integrity, and apoptosis (Jalili‐Firoozinezhad et al., 2018). For this, researchers employed a sophisticated model that integrated human intestinal epithelial cells and endothelial cells within a microfluidic device (Figure 3). This allowed simulation of fluid flow and mechanical deformation, critical for maintaining the functionality in intestinal tissue. The effects of γ‐radiation on villus morphology, barrier function, cellular toxicity, and apoptosis were investigated in this model. The device had two parallel microchannels separated by a porous, flexible, extracellular matrix‐coated membrane lined Caco‐2 cells on one side and human umbilical vein microvascular endothelial cells on the other. Medium was perfused through both channels and mechanical strain was generated by applying cyclic suction through hollow side chambers. The chip accurately mimicked the damage observed in irradiated intestinal tissue in vivo, emphasizing its reliability in replicating human intestinal injury responses. Vascular endothelium was found to mediate radiation‐induced epithelial damage, and the presence of endothelial cells influenced villus morphology, tight junction integrity, and mucin levels in response to radiation exposure (Figure 3). The model serves as a valuable platform for evaluating radiation‐induced cell death, GI acute syndrome, and screening of novel radio‐protective medical countermeasure drugs (Jalili‐Firoozinezhad et al., 2018).

### 3.3 Inflammation

In a study of inflammatory bowel disease (IBD), a complex, multifactorial condition characterized by chronic inflammation of the GI tract (Novak & Mollen, 2015), a gut‐on‐a‐chip model incorporated primary human intestinal epithelial cells derived from patient biopsies and monocyte‐derived macrophages (Figure 3) (Beaurivage et al., 2020). For this, intestinal crypts were first isolated from biopsies and cultured in Matrigel to form three‐dimensional organoids. After stabilization and expansion, the organoids were dissociated and seeded into the upper channel of the microfluidic device containing extracellular matrix gel. This allowed for maintenance of organoid structure while providing a controlled environment for nutrient and fluid flow. The use of primary human intestinal cells enhanced the physiological relevance of the model, while the inclusion of macrophages allowed for exploration of epithelial–immune interactions, key to understanding GI function. The study also described the use of transcriptomics and gene ontology enrichment, showcasing the possibility for omics analyses in these systems that demonstrated gene expression patterns closely resembling those of the adult human colon in vivo, thus affirming the physiological relevance of the model (Beaurivage et al., 2020).

Gut‐specific macrophages are unique in maintaining immune tolerance while protecting against pathogens, unlike other tissue‐resident macrophages, as they exhibit low inflammatory responses to commensal bacteria while efficiently clearing apoptotic cells and microbial fragments (Muller et al., 2020). This study relied on generic macrophages that lack gut‐specific properties. Future advancements should focus on deriving macrophages from intestinal tissue and validating function through transcriptomic profiling to improve disease modeling and drug development (Beaurivage et al., 2020).

### 3.4 A microfluidic platform for neuron‐epithelial coculture

Interactions between intestinal epithelial cells and enteric neurons influence gut motility, secretion, and overall health (Chanpong et al., 2022; Latorre et al., 2016). Traditional in vitro models have faced challenges in replicating the dynamic and multifaceted environment of the gut, thereby limiting our understanding of neuroepithelial communication (Bermudez‐Brito et al., 2013; Schweinlin et al., 2016). Most existing models rely on static cell cultures, which fail to capture the physiological relevance of primary cells or the intricate cellular interactions observed in vivo and do not allow for real‐time observation of neuroepithelial contacts to facilitate the study of their functional implications (Veszelka et al., 2018).

To address these limitations, one study utilized a two‐cell culture setup to mimic the neuroepithelial interactions within the GI tract (de Hoyos‐Vega et al., 2023). The model integrated enteric neurons with epithelial cells to study their interplay in GI disorders (Figure 3). The microfluidic platform was designed with two polydimethylsiloxane layers: a flow layer and a valve layer. The bottom flow layer contained two parallel cell culture compartments connected by microgrooves, which guided neural processes, along with a valve‐controlled port to enable efficient cell injection. The microgrooves specifically directed enteric neurites into the epithelial compartment, promoting the formation of neuroepithelial connections. The device enabled high‐resolution imaging and preserved the phenotypes of intestinal epithelial cells and neurons, allowing for neuroepithelial connections to form under physiologically relevant conditions over long periods (several days).

The study analyzed the extension of neuronal projections into the epithelial compartment and their subsequent contact with epithelial cells to assess neuronal connectivity. Additionally, the integrity of the epithelial barrier was evaluated through markers like ZO‐1 and E‐cadherin and by measuring transepithelial electrical resistance to gauge tight junction integrity. Cell viability and proliferation are assessed through live/dead assays to determine the health of both epithelial cells and neurons, alongside quantifying morphological changes induced by neuron–epithelial interactions. The study successfully developed a novel microfluidic device that allows for the coculture of enteric neurons and intestinal epithelial cells, maintaining their phenotypes and facilitating meaningful neuroepithelial interactions. This platform provides a valuable tool for dissecting the structure and function of neuroepithelial connections in the gut and other organs, advancing our understanding of gut–brain interactions in health and disease (de Hoyos‐Vega et al., 2023).

### 3.5 Interorgan interactions

**FIGURE 4:**
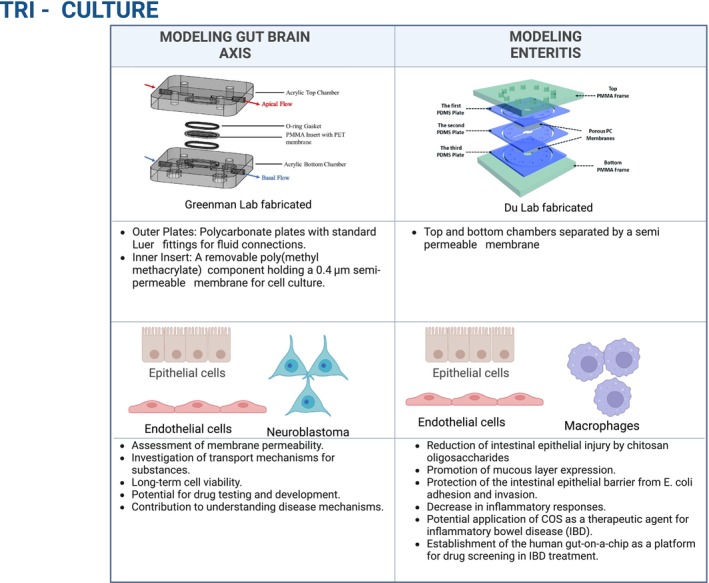
Tri‐culture gut‐on‐a‐chip platforms for studying complex interactions within the gut microenvironment. The figure illustrates tri‐culture systems for modeling the gut‐brain axis and enteritis. The gut‐brain axis model uses epithelial cells, endothelial cells, and neuroblastoma cells in a dual‐flow chamber setup with shear stress to study membrane permeability, transport mechanisms, disease mechanisms, and drug testing. The enteritis model incorporates epithelial cells, endothelial cells, and macrophages in a three‐layer system with flow and cyclic mechanical stretch to investigate intestinal epithelial injury, inflammatory responses, and the therapeutic potential of chitosan oligosaccharides for inflammatory bowel disease (Baldwin et al., 2023; Jing et al., 2022). Created in BioRender. Tang, Y. (2025) https://BioRender.com/y53m745. Figure of devices reproduced from Baldwin et al. (2023), Jing et al.(2022 ) with permission using Creative Common License. View a copy of license: https://creativecommons.org/licenses/by/4.0/. This figure is reproduced with permission (Baldwin et al.,(2023); Jing et al.(2022) This figure is licensed under a Creative Commons Attribution 4.0 International License. To view a copy of this license, visit http://creativecommons.org/licences/by/4.0/.

One study developed a microfluidic platform that integrated gut epithelial and brain endothelial cells to facilitate the investigation of interorgan communication (Figure 1, 2, 3, 4) (Kim et al., 2021). The primary objectives were to develop a modular microfluidic chip for coculture of gut and brain cells, investigate the transport of substances such as exosomes across the gut and blood–brain barriers, and explore the effects of microbial byproducts on these barriers. The chip was fabricated using a three‐dimensional printer to create a gut barrier module and a blood–brain barrier module, coculturing gut epithelial and brain endothelial cells. The cells were cocultured in a modular microfluidic chip, allowing them to be adjacent while maintaining separate environments, which facilitated the study of their interactions. Conditions were set and monitored through controlled fluidic flow in the microchannels of the chip, and trans‐endothelial/epithelial electrical resistance was measured through the incorporation of electrodes into the device. Additionally, fluorescence microscopy was employed to visualize exosome transport and evaluate cell viability. The results indicated that the cultured cells maintained good viability throughout the experiments, as confirmed by a Live/Dead assay. Cells were exposed to treatments such as lipopolysaccharide or butyrate to observe changes in barrier function, which was assessed by measuring trans‐endothelial/epithelial electrical resistance. The model provided valuable insights into intercellular communication mechanisms through its innovative coculture system that allowed the simultaneous cultivation of gut epithelial and brain endothelial cells. This design facilitated direct communication between the two cell types and enabled the simulation of in vivo conditions by incorporating fluidic flow, which was essential for studying the physiological processes affecting the transport of signaling molecules, like exosomes, across cell barriers. The ability to control the spatiotemporal environment provided a unique opportunity to study transport phenomena in a physiologically relevant context, enhancing the translatability of research findings to clinical applications.

The study successfully replicated both the gut epithelial and blood–brain barriers and assessed the transport of fluorescently labeled exosomes from the intestinal layer to the blood–brain barrier (Kim et al., 2021).

Additionally, a recent study developed an organ‐on‐chip platform to model drug metabolism along the gut‐liver axis, providing new insights into how drugs absorbed in the intestine undergo metabolic transformation in the liver before reaching systemic circulation. Beyond gut–brain interactions, drug metabolism in the gut has far‐reaching systemic effects, particularly through the gut‐liver axis, which plays a crucial role in first‐pass metabolism, drug bioavailability, and detoxification (Lucchetti et al., 2024). A deeper understanding of interorgan interactions, including the crosstalk between the gut, liver, and other organs, is essential for improving drug efficacy predictions, minimizing adverse effects, and optimizing therapeutic strategies.

## 4 TRI‐CULTURE

Tri‐culture models are models that incorporate three distinct cell types, typically a combination of gut epithelial cells, immune or neuronal cells, and either microbial or endothelial cells, to better mimic the complexity of the gut environment.

Tri‐culture models offer a more complex and physiologically relevant platform for studying the intricate interactions within the gut environment (Figure 4) (Baldwin et al., 2023; Jing et al., 2022). Each step in the progression from cellular mono‐cultures to tri‐culture models adds a layer of complexity and insight that is crucial for understanding the multifaceted nature of GI function. Incorporation of a third cell type, typically immune or neuronal cells, along with gut epithelial, microbial cells, or endothelial cells (Baldwin et al., 2023; Jing et al., 2022) renders the tri‐culture models more representative of the in vivo gut environment and provides a more accurate insight into host–microbiota interactions in health and disease progression (Baldwin et al., 2023; Jing et al., 2022). Tri‐culture microfluidics models are rare due to the complexity of host–microbiota interactions, and we review the existing models below.

### 4.1 Modeling symptoms of enteritis

In a tri‐culture microfluidic chip, intestinal epithelial cells, vascular endothelial cells, and macrophages were cocultured to explore the therapeutic potential of chitosan oligosaccharides, a natural polymer, in alleviating cellular markers of enteritis (Figure 4) (Jing et al., 2022). The study modeled enteritis by introducing pathogenic *Escherichia coli* into the microfluidic microchannel containing the villus epithelium, while macrophages were delivered into the vascular endothelial channel to mimic the inflammatory response typically observed in enteritis. A key feature of the model was its ability to facilitate real‐time monitoring of cellular responses and barrier integrity, thus enabling observation of dynamic changes in the gut environment. By introducing E. coli, the model effectively replicates bacterial interactions and inflammatory responses characteristic of clinical settings. The ability to assess the therapeutic effects of chitosan oligosaccharides on inflammation and epithelial barrier protection provides valuable insights into potential clinical treatments for inflammatory bowel disease. Additionally, the model facilitates the investigation of gut microbiota dynamics in the context of enteritis, further bridging the gap between basic research and clinical implications and offering a platform for drug testing that reflects human biology more accurately than traditional animal studies (Jing et al., 2022). This study represented a significant step forward in the development of in vitro platforms for studying complex multicellular conditions for the evaluation of therapeutic interventions.

#### 4.1.1 The potential and limitations of tri‐culture models

Tri‐culture models represent a significant advancement in simulating gut interactions by allowing observation of multicellular interactions. This includes the incorporation of the gut flora, such as in the case of the introduction of *E. coli* in one study described above (Jing et al., 2022). This opens up possibilities for close investigation of the host–microbiota interactions in multi‐chamber systems capable of regulating individual chamber oxygen gradients and holds the potential for investigation of the microbiota‐gut‐brain axis, a vital area for understanding gut‐brain communication and its implications for related diseases. Current models have yet to address this gap adequately, and few systems support the sustained growth and coculture of anaerobic gut microbiota, a necessary factor for studying the intricate dynamics of this ecosystem. In addition, the design is technically demanding, requiring expertise in microfluidics, cell culture, and device assembly, which can limit their accessibility in certain research contexts (Baldwin et al., 2023). To fully harness the potential of tri‐culture models, further development is essential. Enhancing their capability to model complex biological interactions, including the microbiota‐gut‐brain axis, will be crucial for advancing research in this field.

### 4.2 Modeling the gut‐brain axis in microfluidic device

A micro physiological system that integrated multicellular components of the gut and brain provided a step forward from a bi‐culture platform, allowing investigation of multicellular interactions. By employing a dual‐flow tissue perfusion device, the model maintained cell viability and functionality over extended periods, enabling long‐term studies of the gut‐brain axis (Baldwin et al., 2023). The primary aim was to explore the transport of bacterial extracellular vesicles, which play a role in mediating communication between the gut microbiota and the central nervous system (Baldwin et al., 2023). The tri‐culture system cocultured human colorectal adenocarcinoma cell lines (Caco‐2 and HT29‐MTX‐E12), Human Umbilical Vein Endothelial Cells (HUVECs), and human neuroblastoma cells (SH‐SY5Y) (Baldwin et al., 2023). The device was designed with two separate chambers representing the gut and the brain, connected and operated under modular flow conditions (Figure 4). Epithelial cells were seeded in the apical side, and endothelial cells were cocultured on the basal side of the chamber representative of the luminal and serosal sides, respectively. Neurons were cultured in a separate chamber representative of the nervous system. Cell viability was maintained for a minimum of 7 days under perfusion, with cells reaching 100% confluency and remaining viable throughout the experimental timeline. In this model, the transport of bacterial extracellular vesicles across the epithelial‐endothelial layer was successfully measured, as was their uptake by the neuronal cells in the brain chamber, demonstrating the model's capacity to mimic the transport of gut‐derived substances across the gut‐brain barriers. This model tackled several limitations of existing microfluidic models and provided a flexible and robust system that is accessible to a broader range of researchers (Baldwin et al., 2023). The study highlights the innovative use of connected perfusion systems to replicate complex gut–brain interactions, showcasing the model's versatility in studying the transport of extracellular vesicles across epithelial and endothelial barriers.

## 5 CONCLUSIONS

Gut‐on‐a‐chip platforms have significantly advanced gastrointestinal research and drug development, demonstrating their potential as transformative tools. These systems have enabled the elucidation of neuroepithelial interactions, enhancing our understanding of gut‐brain communication and its role in gastrointestinal disorders (de Hoyos‐Vega et al., 2023). They have also been instrumental in uncovering therapeutic potential, such as the novel finding that chitosan oligosaccharides can inhibit the onset and progression of enteritis, suggesting their promise in treating inflammatory bowel disease (Jing et al., 2022). Additionally, these platforms have facilitated studies on the impact of mycotoxins by replicating physiological flow dynamics, offering insight into route‐dependent toxicity and its effects on intestinal barrier integrity (Pöschl et al., 2023).

Recent innovations, such as the integration of induced pluripotent stem cells (iPSCs), further elevate the physiological relevance of these models. iPSCs allow for the differentiation of diverse intestinal cell types, support personalized medicine, and improve the longevity of culture systems (Moerkens et al., 2024). However, challenges remain in harnessing their full potential, including the complexity of differentiation protocols, the need for finely tuned growth factor gradients, and the difficulty in sustaining long‐term cultures (Hall & Bendtsen, 2023).

Despite these advancements, widespread adoption of gut‐on‐a‐chip models in laboratory settings remains limited due to several challenges. These include technical complexity, difficulties in maintaining long‐term cell viability, and a lack of standardized protocols. Additionally, the reliance on custom‐fabricated devices increases costs and time investment, emphasizing the urgent need for more commercially available and accessible platforms to expand the use of gut‐on‐a‐chip technology in research and drug development.

Overall, gut‐on‐a‐chip technology represents a critical bridge between traditional 2D cell cultures and animal models (Table 1). By recreating the dynamic microenvironment of the human gut, including peristaltic motion, fluid flow, nutrient transport, and microbial colonization, these platforms continue to drive forward our ability to model human gut physiology and disease. Their ongoing evolution holds significant promise for uncovering disease mechanisms and accelerating the development of novel therapeutics.

**TABLE 1:** Summary of gut on a chip platforms used for gastrointestinal studies.

Group	Culture conditions	Purpose	Design	Cells	Mechanical simulation	Availability
Guo et al. (2018)	Cellular Mono‐culture	Testing drug metabolism of oral drugs	Three layers: a microfluidic channel, a Nitrocellulose membrane, a PDMS layer	Human colorectal adenocarcinoma cell line (Caco‐2)	Flow (shear stress)	Lab fabricated
Pöschl et al. (2023)		Testing of mycotoxin	Three microfluidic channels separated by membrane free phase guide	Human colorectal adenocarcinoma cell line (Caco‐2)	Gravity induced flow (rocker)	Commercially available
Villenave et al. (2017)		Modeling enterovirus infection	Two hollow microchannels separated by a porous membrane	Human colorectal adenocarcinoma cell line (Caco‐2)	Flow (shear stress) cyclical mechanical stretch (peristalsis)	Lab fabricated
Shim et al. (2017)		Replicating three‐ dimensional villi structure	Three layers, a slide glass and a polyester membrane	Human colorectal adenocarcinoma cell line (Caco‐2)	Flow (shear stress)	Lab fabricated
Wang et al. (2023)		Modeling transport	Top and bottom microchannels and middle porous membrane	Human colorectal adenocarcinoma cell line (Caco‐2)	Flow (shear stress) cyclical mechanical stretch (peristalsis)	Lab fabricated
Jalili‐Firoozinezhad et al. (2018)	Bi culture	Modeling radiation injury induced cell death	Two channels separated by a porous membrane	Human colorectal adenocarcinoma cell line (Caco‐2), human umbilical vein microvascular endothelial cells	Flow (shear stress) Cyclic stretching through vacuum chambers to mimic peristalsis	Lab fabricated
Beaurivage et al. (2020)		Modeling key aspects of Inflammatory Bowel Disease	Three microfluidic channels separated by membrane free phase guide	Intestinal epithelial cells derived from human intestinal organoids monocyte‐derived macrophages	Gravity induced flow (rocker)	Commercially available
Kim et al. (2021)		Modeling transport	Two separate compartments separated by a porous membrane	Human colorectal adenocarcinoma cell line (Caco‐2), either murine brain endothelial cell line (bEnd.3) or primary human brain microvascular endothelial cells (hBMECs)	Flow (shear stress)	Lab fabricated
de Hoyos‐Vega et al. (2023)		Modeling the gut‐brain axis	Two layers: flow and valve layer	human colon organoids, enteric neurons		Lab fabricated
Kim et al. (2012)		Microbiome culture with intestinal layer	Two microchannels were separated by a porous membrane	Human colorectal adenocarcinoma cell line (Caco‐2), Lactobacillus rhamnosus GG	Flow (shear stress) cyclical mechanical stretch (peristalsis)	Lab fabricated
Baldwin et al. (2023)	Tri‐culture	Modeling the gut‐brain axis	Outer Plates: Polycarbonate plates with standard Luer fittings for fluid connections. Inner Insert: A removable poly(methyl methacrylate) component holding a 0.4 μm semi‐permeable membrane for cell culture. Dual‐Flow Channels	Human colorectal adenocarcinoma cell lines (Caco‐2, HT29‐MTX‐E12), Human Umbilical Vein Endothelial cells (HUVECS) and human neuroblastoma SH‐SYSY	Flow (shear stress)	Lab fabricated
Jing et al. (2022)		Modeling enteritis	Top and bottom chambers separated by a semi permeable membrane	Human colorectal adenocarcinoma cell line (Caco‐2), human macrophage cells, human umbilical vein endothelial cells (HUVECs)	Flow (shear stress) cyclical mechanical stretch (peristalsis)	Lab fabricated

## 6 PERSPECTIVES

Multi‐culture organ‐on‐a‐chip models have the potential to transform biomedical research by reducing costs, minimizing reliance on animal models, and enhancing the translational relevance of in vitro studies. Their ability to closely replicate organ physiology will enable more accurate predictions of drug efficacy and disease progression. Expanding the availability of commercially produced devices would address the problems of time and costs associated with custom fabrication and make them more accessible to a broader range of laboratories to foster widespread adoption.

However, despite these advancements, extrapolating data from gut‐on‐a‐chip models to in vivo and clinical applications remains a significant challenge. While these systems simulate key physiological parameters, critical gaps persist in mimicking the full complexity of the gastrointestinal environment, including immune responses, host–microbe interactions, and interorgan communication. To improve clinical relevance, future gut‐on‐a‐chip platforms must integrate biomechanical cues, dynamic microbiome modeling, and multi‐organ interactions. Moreover, the regulatory landscape must evolve to incorporate organ‐on‐a‐chip models as standardized preclinical tools for drug development.

To enhance their utility in drug discovery, gut‐on‐a‐chip models must improve predictive accuracy for human physiology by incorporating drug metabolism, immune–gut interactions, and systemic responses. The integration of liver, kidney, and blood–brain barrier models will enable comprehensive drug testing, while patient‐specific iPSC‐derived gut models could advance personalized medicine. Expanding these platforms with long‐term culture capabilities, patient‐derived organoids, and real‐time biosensing will further strengthen their role in translational research. However, achieving widespread adoption requires addressing standardization challenges and regulatory approval. Establishing validated biomarkers, high‐throughput screening methods, and multi‐organ system integration will be critical for pharmaceutical applications.

By addressing these limitations and enhancing model complexity, gut‐on‐a‐chip platforms can bridge the gap between preclinical research and clinical applications, offering a cost‐effective, ethical, and human‐relevant alternative to traditional drug testing methods. As these technologies advance, they hold the potential to revolutionize drug discovery and precision medicine, leading to more accurate predictions of drug efficacy and safety while reducing reliance on animal testing and conventional cell culture models.

## 7 FUNDING INFORMATION

None.

## 8 ETHICS STATEMENT

This review paper is based solely on previously published literature and does not include any new experiments involving human subjects or animals. As such, ethical approval was not required.
